# Neuroendocrine Tumor Metastases to the Breast Mimic Breast Primary Carcinoma: Mammography and Multimodality US Assessment in Challenging Differential Diagnosis

**DOI:** 10.3390/diagnostics15070860

**Published:** 2025-03-28

**Authors:** Francesco Marcello Aricò, Antonio Portaluri, Francesca Catanzariti, Elvira Condorelli, Demetrio Aricò, Mariagiovanna Zagami, Emilia Magliolo, Sara Monforte, Maria Adele Marino

**Affiliations:** 1 Diagnostic and Interventional Radiology Unit, BIOMORF Department, University Hospital “Policlinico G. Martino”, 98124 Messina, Italy; 2 Department of Nuclear Medicine, Humanitas Oncological Centre of Catania, 95125 Catania, Italy; 3 Pathology Unit, Ospedale “San Vincenzo”, 98039 Taormina (ME), Italy; 4 UOC Radiologia Sezione di Senologia, Ospedale “San Vincenzo”, 98039 Taormina (ME), Italy

**Keywords:** breast metastases, neuroendocrine tumors (NETs), 68Ga-DOTATOC PET-CT, somatostatin receptor imaging, multimodality ultrasound, differential diagnosis, pancreatic NET, shear wave elastography (SWE), contrast-enhanced ultrasound (CEUS), misdiagnosis

## Abstract

Metastases to the breast from non-mammary malignancies are rare, accounting for 0.1–5% of all breast malignancies. Neuroendocrine tumors (NETs) rarely metastasize to the breast. PET-CT somatostatin receptor imaging plays a pivotal role in the staging and follow-up of NETs, leveraging tracers like 68Ga-DOTATOC that bind to somatostatin receptors (SSTRs) expressed on tumor cells. While both primary and metastatic NETs express SSTRs, primary breast tumors may also exhibit an uptake of 68Ga-somatostatin analogs, making the differential diagnosis between primary breast tumors and neuroendocrine metastases challenging. Additionally, imaging characteristics of breast metastases from NETs are poorly documented in the literature, posing a diagnostic challenge that extends to pathology, particularly when in the absence of clinical suspicion. Misdiagnosis in such cases can lead to inappropriate therapeutic interventions. We report the case of a 75-year-old female patient with a history of pancreatic NET who presented to our breast clinic for further evaluation of a breast mass after a PET-CT scan revealed moderate 68Ga-DOTATOC uptake. Multimodality breast examination, including mammography and multiparametric US with B-mode, Color Doppler, Strain Elastography (SE), Shear Wave Elastography (SWE), and contrast-enhanced US (CEUS), was performed. Following a core biopsy, the lesion underwent surgical excision, revealing the diagnosis of NET metastasis. This case highlights a rare instance of neuroendocrine tumor metastasis to the breast, assessed using various ultrasound techniques, with detailed imaging and quantitative analysis. The comprehensive multimodal assessment contributes to the limited body of literature and provides elements for the differential diagnosis of a rare breast lesion that should always be considered in the presence of a known primary NET.

**Figure 1:**
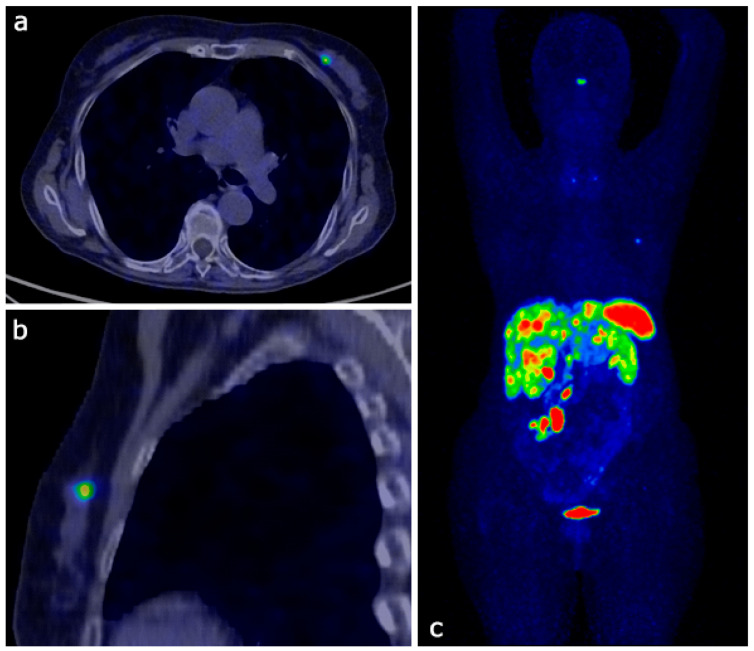
A 75-year-old female patient with a clinical history of stage IV neuroendocrine tumor (NET) of pancreatic origin, with metastases to lymph nodes and liver, came to our breast clinic for further diagnostic evaluation following a PET/CT scan. 68Ga-DOTATOC PET/CT Transaxial (**a**) and sagittal (**b**) images show a focal faint uptake in the upper inner quadrant (UIQ) of the left breast (KS = 2); the MIP image (**c**) shows intense peptide uptake in the midgut, lymph nodes, pancreas, and liver (KS = 3-4). KS or Krenning score: 0 = no uptake; 1 = very low uptake; 2 = uptake less than/equal to liver; 3 = uptake greater than liver; 4 = uptake greater than spleen. Metastases to the breast from non-mammary primary malignancies are rare, accounting for approximately 0.1–5% of all breast malignancies, with no clear consensus on the exact prevalence [1,2,3]. The most common cancers metastasizing to the breast include malignant melanoma, lymphoma, lung cancer, and, in men, prostate cancer [1,2]. Neuroendocrine tumors (NETs) can also spread to the breast, though this occurrence is particularly uncommon [2,4,5,6,7]. PET-CT somatostatin receptor imaging plays a crucial role in the staging and follow-up of NETs. This technique leverages the high affinity of specific tracers, such as the 68Ga-labeled somatostatin analogs, for somatostatin receptors (SSTRs) expressed on tumor cell surfaces. Various radiolabeled somatostatin analogs, including 68Ga-DOTATOC, 68Ga-DOTANOC, and 68Ga-DOTATATE, have been developed for neuroendocrine tumor imaging, differing in their affinity for specific somatostatin receptor subtypes [8,9]. Both primary and metastatic NETs typically express SSTRs [6,10]; however, studies have shown that primary breast tumors may also exhibit avidity for 68Ga-somatostatin analogs [11,12,13]. This presents a diagnostic challenge in distinguishing between primary breast lesions and metastatic lesions originating from NETs. Moreover, imaging features of NETs metastases to the breast have not been extensively explored [14,15]. The identification of metastatic NET can also be particularly challenging for pathologists, especially when relying on biopsy specimens unaware of clinical information. This diagnostic complexity increases the risk of misdiagnosis, potentially leading to sub-optimal therapeutic strategies [16]. The high affinity of 68Ga-somatostatin analogs for SSTRs has significantly transformed the imaging NETs. In recent years, scintigraphy (single-photon emission computed tomography, SPECT/CT) using 111In-Pentreotide and 99mTc-Octreotide has been largely replaced with 68Ga-DOTATOC-SSA PET/CT, due to its superior diagnostic efficiency [4,6]. 68Ga-DOTATOC physiologically distributes in organs that naturally express high levels of somatostatin receptors (SSTRs). The Krenning score is a semiquantitative grading system assessing SSTR expression in NETs on scintigraphy and PET imaging, with higher scores (≥3) indicating strong receptor expression. Its determination requires comparison to the liver and spleen [17]. Both primary neuroendocrine tumors (NETs) and their metastases generally demonstrate a high expression of these receptors [6,10,18]. The most frequent sites of NET metastases include the lymph nodes, liver, and lungs; however, less common, metastatic sites such as the bone, orbita, heart, Virchow’s lymph node, and breast have been reported [4]. Wedin et al. reported pathological 68Ga-DOTATOC uptake in the breast in 1.5% of patients (18/1171) with a history of NET [7]. According to Glazebrook et al., breast metastases from gastrointestinal NETs accounted for 0.1% of 7000 breast malignancies studied [15]. Cabrero et al. reported an even rarer occurrence, with breast metastases from ileal NETs comprising only 0.009% of 10,650 breast malignancies [19]. On the other hand, it is important to note that primary breast cancer can also exhibit 68Ga-somatostatin analog avidity. Studies report that up to 20% of breast cancers exhibit neuroendocrine differentiation, characterized by SSTR overexpression and synaptophysin/chromogranin positivity [11]. Therefore, all such breast lesions should undergo extended breast examination, including histopathological analysis, to determine the exact nature of the lesion.

**Figure 2:**
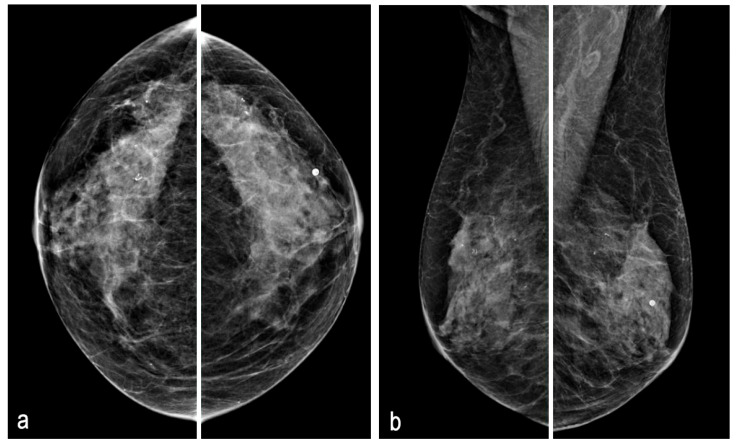
Mammographic CC (**a**) and MLO (**b**) views revealed heterogeneously dense breast, which may obscure small masses (ACR category c). However, in the UIQ, the nodular uptake reported on the PET-CT was identified as a 10 mm mass with an irregular shape and partially obscured margin. No prior mammographic studies were available for comparative purposes. No suspicious microcalcifications were observed. In other studies, metastases have been reported on mammography as well circumscribed, round, or oval masses without calcifications. Less commonly, they may present as spiculated masses [14,15].

**Figure 3:**
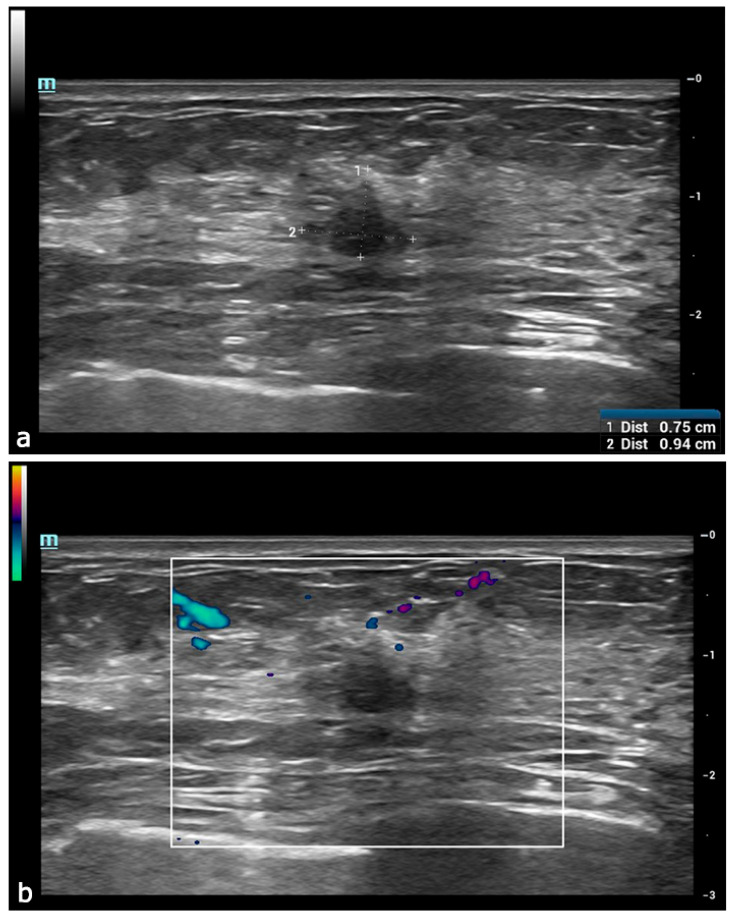
Subsequently, the patient underwent a multiparametric US examination, which included B-mode, Color Doppler, Strain Elastography (SE), Shear Wave Elastography (SWE), and contrast-enhanced ultrasound (CEUS). B-mode ultrasound of the UIQ of the left breast (**a**) revealed a hypoechoic irregular-shaped lesion with microlobulated margins and anti-parallel orientation, measuring approximately 9 × 7 mm. No intralesional vascular signals were found at the Color Doppler evaluation (**b**). No other suspicious lesions were detected, identifying the lesion as solitary. Given these findings, benign lesions were considered less likely in the differential diagnosis, with the main differential being between a primary breast lesion and a metastatic tumor from a NET, despite the latter being extremely rare. The lesion was classified as BI-RADS category 4C, indicating a moderate suspicion of malignancy and warranting a US-guided core biopsy.

**Figure 4:**
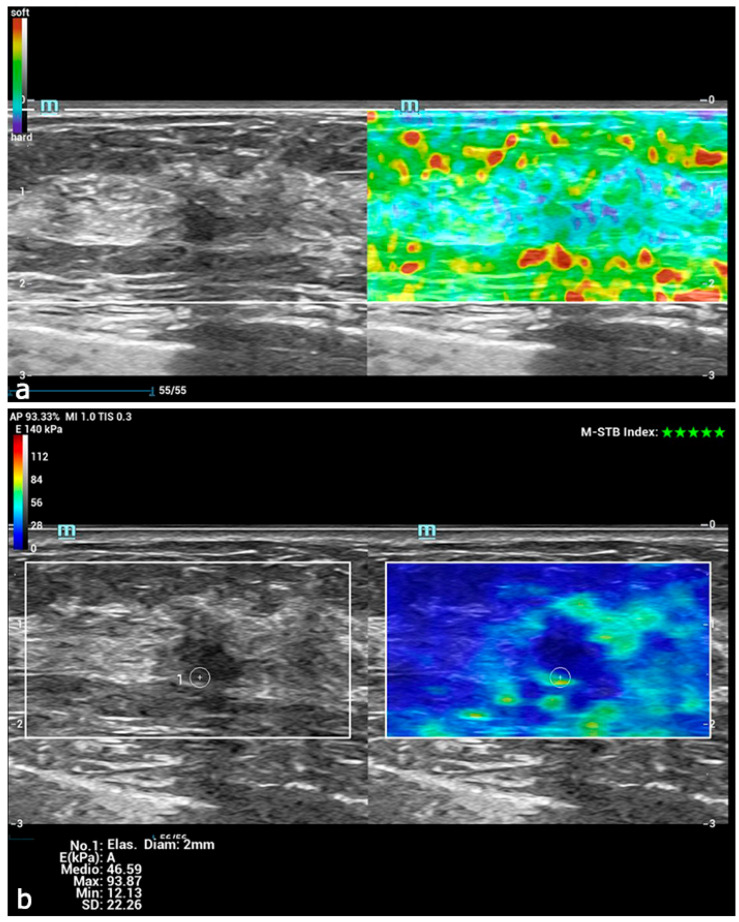
Strain Elastography (SE) (**a**) and Shear Wave Elastography (SWE) (**b**) assessments were performed during the same ultrasound examination. SE displayed a green color on the colorimetric map, indicating an intermediate degree of stiffness. SWE measured an average stiffness of 46 kPa in the stiffest part of the lesion, which is a value significantly lower than those reported in the literature for differentiating benign and malignant lesions [20]. The available literature on the ultrasound imaging of breast neuroendocrine tumor (NET) metastases is limited and does not provide reliable criteria with which to distinguish metastatic lesions from primary breast tumors. However, reported cases describe a variety of imaging features. Most lesions appeared as hypoechoic, irregular masses, while others were isoechoic to surrounding fat with a thin echogenic halo. In some instances, lesions exhibited increased acoustic transmission, suggesting possible complex cysts. None of the reported cases demonstrated posterior acoustic shadowing. Color Doppler evaluation frequently revealed marked vascularity, though some lesions showed no detectable flow on power Doppler. Rigid appearance at Strain Elastography has also been reported [14,15].

**Figure 5:**
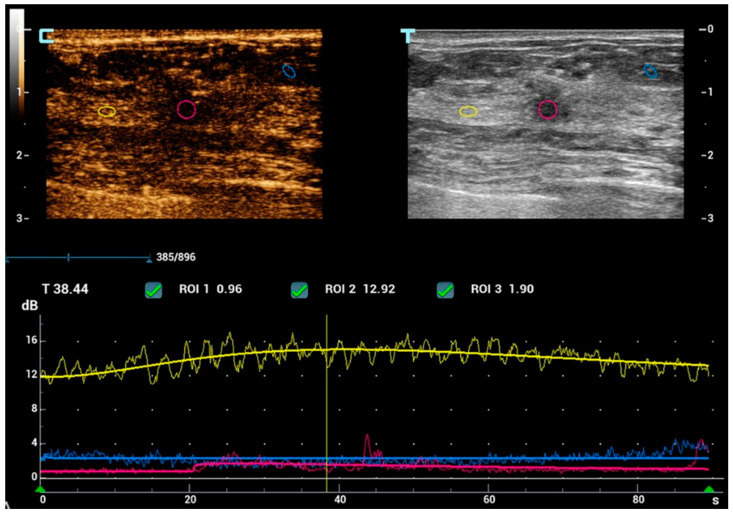
After obtaining informed consent, CEUS was also performed as part of the multiparametric US evaluation, with an intravenous infusion of 2 mL of SonoVue (Bracco Inc., Milan, Italy). Throughout the dynamic study, the nodule did not demonstrate contrast uptake, as confirmed by the time-intensity curve (TIC). The figure illustrates the placement of three ROIs: a pink ROI within the lesion, a yellow ROI in the adjacent fibro-glandular breast tissue, and a blue ROI in the surrounding adipose tissue. TIC analysis demonstrates the absence of enhancement within the nodule. To the best of our knowledge, this is the first reported case of NET metastases to the breast evaluated with an extended breast imaging study, including both mammographic evaluation and multiparametric ultrasound assessment with CEUS. The qualitative assessment did not reveal typical radiological features suggestive of malignancy, such as heterogeneous and centripetal enhancement, which are widely recognized in the literature as indicative of malignant behavior [21]. The lesion demonstrated nonspecific suspicious features; however, no distinct imaging characteristics have been reported in the literature to reliably differentiate a primary pathological breast lesion from a NET metastasis. These two entities may appear indistinguishable both on imaging and in their uptake of a 68Ga-somatostatin analog, underscoring the necessity of diagnostic confirmation through core biopsy [5,6,7,12,13,15,22].

**Figure 6:**
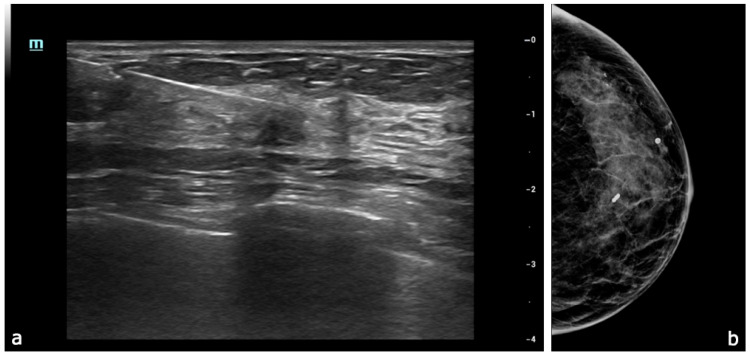
Following imaging assessments, five specimens were obtained using a 14G × 10 cm automatic biopsy needle (**a**). A titanium marker was placed at the biopsy site, and its position was confirmed via monolateral mammography in mediolateral (ML) and craniocaudal (CC) views (**b**), showing the marker in the site of the lesion. The histological examination revealed a B5 lesion, which was initially classified as an invasive ductal carcinoma (IDC). The immunohistochemical profile showed negativity for androgen receptor (AR), estrogen receptor (ER), progesterone receptor (PGR), and HER2, with a Mib-1 proliferation index of less than 10%, and p63 negativity.

**Figure 7:**
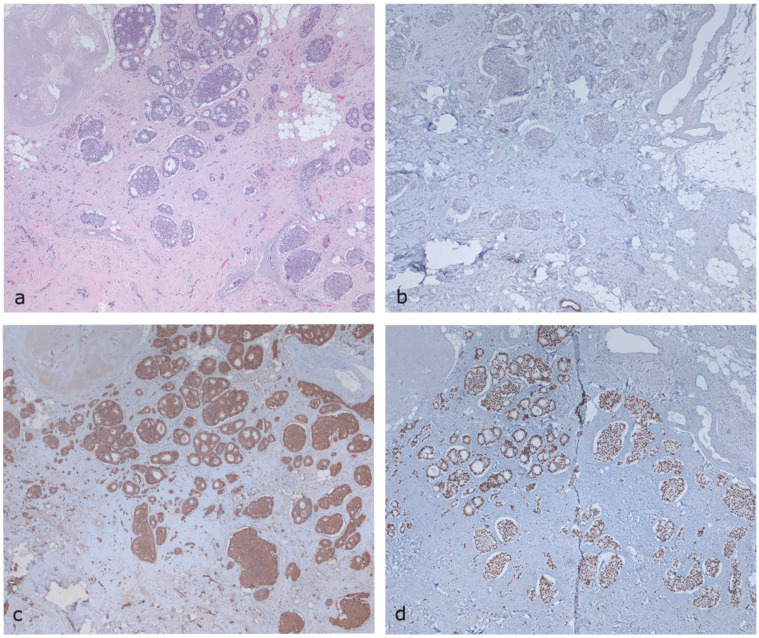
The patient subsequently underwent left quadrantectomy of the upper inner quadrant and sentinel lymph node biopsy (SLNB). The latter was negative for neoplastic localization. The postoperative histological examination showed the neuroendocrine nature of the lesion, documenting a 6 mm, grade 2 (G2), neuroendocrine tumor as a secondary lesion from a gastrointestinal NET. The imaging included the following: (**a**) Hematoxylin and Eosin (H&E) staining, (**b**) GATA3 immunohistochemistry (GATA3), (**c**) chromogranin immunohistochemistry (CgA), and (**d**) CDX2 immunohistochemistry (CDX2), documenting neoplastic proliferation with an organoid growth pattern, in nests, insulae, and pseudo-rosettes, consisting of relatively monomorphic elements with round or oval nuclei and salt-and-pepper chromatin. Immunohistochemical investigation revealed positivity for synaptophysin, chromogranin, CK19, CK8/18, and CDX2 and negativity for CK7, GATA3, ER, PGR, HER2, TTF-1, and PAX-8. The proliferation index, assessed using Ki-67, was approximately 5% of the neoplastic cellularity. The immunophenotypic profile, also considering the clinical history, primarily suggests a secondary localization from a NET. This case suggests that despite the a prior diagnosis of a primary NET, misdiagnosis may easily occur [16]. In the absence of accurate clinical context, diagnosing metastatic NET can be challenging for pathologists, particularly on biopsy specimens. In fact, while most patients with breast metastases have a known history of primary NET, this information could not be accessible to pathologists during biopsy evaluation. In this scenario, a frequent factor of misdiagnosis is a communication failure between radiologists and pathologists [23,24]. In the study by Perry et al., 44% of breast metastases originating from NETs were misdiagnosed as primary breast carcinomas [16]. Other studies have demonstrated the significant tendency of metastatic NETs to closely mimic primary breast malignancies [14,25,26,27]. The primary reasons for misdiagnosis can be attributed to an inaccurate assessment of the clinical context, the failure in communication between radiologists and other specialists, the rarity of breast metastases from neuroendocrine tumors, and the morphological similarity to invasive breast carcinomas. Misdiagnosis can lead to an inappropriate therapeutic approach, potentially resulting in overtreatment. In particular, when metastatic NETs are mistaken for primary breast carcinoma, patients may undergo unnecessary aggressive treatments, including mastectomy and axillary lymph node dissection, which provide no survival benefit in the context of metastatic disease. This misclassification may not only subject patients to unwarranted surgical morbidity but also delay the initiation of appropriate systemic therapy tailored to the underlying neuroendocrine malignancy [16]. To the best of our knowledge, the management of breast metastases from NETs lacks specific guidelines [10,28]. Surgery, such as lumpectomy without axillary dissection, could be considered, while somatostatin analogs may be used for their antiproliferative/anti-hormonal effects. In contrast, primary breast carcinoma typically requires a more radical therapeutic approach. In such cases, overtreatment such as mastectomy, unnecessary radiation, axillary lymph node biopsy, or systemic therapy for breast cancer could be avoided [27]. In conclusion, data on the imaging characteristics of NET metastases to the breast are limited, and imaging findings are often nonspecific and challenging to differentiate from primary breast carcinoma. This is the first documented case providing a comprehensive breast imaging study, incorporating both mammographic evaluation and multiparametric ultrasound examination, including CEUS. In case of a newly detected breast lesion in a patient with a history of gastrointestinal NET, the possibility of breast metastasis should always be considered, despite its rarity. Core biopsy plays a pivotal role in achieving an accurate diagnosis. Close collaboration between radiologists, oncologists, and pathologists, along with a thorough assessment of the patient’s clinical history, is essential to prevent misdiagnosis and ensure appropriate management. A multidisciplinary approach fosters accurate diagnosis, optimizes treatment strategies, and ultimately improves patient outcomes.

## Data Availability

Data are contained within the article.

## References

[1] Vergier B., Trojani M., De Mascarel I., Coindre J., Le Treut A. (1991). Metastases to the breast: Differential diagnosis from primary breast carcinoma. J. Surg. Oncol..

[2] Feder J.M., de Paredes E.S., Hogge J.P., Wilken J.J. (1999). Unusual Breast Lesions: Radiologic-Pathologic Correlation. RadioGraphics.

[3] Li J., Wahab R., Brown A.L., Guarnieri B., Lewis K., Mahoney M.C., Vijapura C. (2023). Extramammary Metastases to the Breast. RadioGraphics.

[4] Naswa N., Sharma P., Kumar R., Malhotra A., Bal C. (2013). Usual and Unusual Neuroendocrine Tumor Metastases on 68Ga-DOTANOC PET/CT. Clin. Nucl. Med..

[5] Crona J., Granberg D., Norlén O., Wärnberg F., Stålberg P., Hellman P., Björklund P. (2013). Metastases from Neuroendocrine Tumors to the Breast Are More Common than Previously Thought. A Diagnostic Pitfall?. World J. Surg..

[6] La Rosa S., Casnedi S., Maragliano R., Goyault G., Weber J.-C., Louis B., Schlund E., Sessa F. (2015). Breast Metastasis as the First Clinical Manifestation of Ileal Neuroendocrine Tumor. A Challenging Diagnosis with Relevant Clinical Implications. Endocr. Pathol..

[7] Wedin M., Janson E.T., Wallin G., Sundin A., Daskalakis K. (2024). Prevalence of metastases outside the liver and abdominal lymph nodes on 68Ga-DOTATOC-PET/CT in patients with small intestinal and pancreatic neuroendocrine tumours. J. Neuroendocr..

[8] Wild D., Bomanji J.B., Benkert P., Maecke H., Ell P.J., Reubi J.C., Caplin M.E. (2013). Comparison of 68Ga-DOTANOC and 68Ga-DOTATATE PET/CT Within Patients with Gastroenteropancreatic Neuroendocrine Tumors. J. Nucl. Med..

[9] Mirzaei S., Revheim M.-E., Raynor W., Zehetner W., Knoll P., Zandieh S., Alavi A. (2019). 64Cu-DOTATOC PET-CT in Patients with Neuroendocrine Tumors. Oncol. Ther..

[10] Sundin A., Arnold R., Baudin E., Cwikla J.B., Eriksson B., Fanti S., Fazio N., Giammarile F., Hicks R.J., Kjaer A. (2017). ENETS Consensus Guidelines for the Standards of Care in Neuroendocrine Tumors: Radiological, Nuclear Medicine and Hybrid Imaging. Neuroendocrinology.

[11] Balma M., Liberini V., Racca M., Laudicella R., Bauckneht M., Buschiazzo A., Nicolotti D.G., Peano S., Bianchi A., Albano G. (2022). Non-conventional and Investigational PET Radiotracers for Breast Cancer: A Systematic Review. Front. Med..

[12] Yamaga L.Y.I., Wagner J., Funari M.B.d.G. (2017). 68Ga-DOTATATE PET/CT in Nonneuroendocrine Tumors. Clin. Nucl. Med..

[13] Ambinder E.B., Werner R.A., Rowe S.P. (2020). Incidental primary breast cancer detected on surveillance 68Ga-DOTATATE PET/CT in a patient with metastatic neuroendocrine carcinoma. Radiol. Case Rep..

[14] Adams R.F., Parulekar V., Hughes C., Kadour M.J., Talbot D. (2009). Radiologic Characteristics and Management of Screen-Detected Metastatic Carcinoid Tumor of the Breast: A Case Report. Clin. Breast Cancer.

[15] Glazebrook K.N., Jones K.N., Dilaveri C.A., Perry K., Reynolds C. (2011). Imaging features of carcinoid tumors metastatic to the breast. Cancer Imaging.

[16] Perry K.D., Reynolds C., Rosen D.G., Edgerton M.E., Albarracin C.T., Gilcrease M.Z., A Sahin A., Abraham S.C., Wu Y. (2011). Metastatic neuroendocrine tumour in the breast: A potential mimic of in-situ and invasive mammary carcinoma. Histopathology.

[17] Park S.Y., Parihar A.S., Bodei L., Hope T.A., Mallak N., Millo C., Prasad K., Wilson D., Zukotynski K., Mittra E. (2021). Somatostatin Receptor Imaging and Theranostics: Current Practice and Future Prospects. J. Nucl. Med..

[18] Reubi J.C., Krenning E. (1992). In Vitro Detection of Somatostatin Receptors in Human Tumors. Metabolism.

[19] Cabrero I., Álvarez M., Montiel D., Tavassoli F. (2003). Metastases to the breast. Eur. J. Surg. Oncol. EJSO.

[20] Youk J.H., Gweon H.M., Son E.J., Han K.H., Kim J.-A. (2013). Diagnostic value of commercially available shear-wave elastography for breast cancers: Integration into BI-RADS classification with subcategories of category 4. Eur. Radiol..

[21] Ito T., Manabe H., Kubota M., Komoike Y. (2024). Current status and future perspectives of contrast-enhanced ultrasound diagnosis of breast lesions. J. Med. Ultrason..

[22] Guirguis M.S., Adrada B.E., Surasi D.S.M., Dryden M.J. (2020). 68Ga-DOTATATE Uptake in Primary Breast Cancer. Clin. Nucl. Med..

[23] Thomassin-Naggara I., Kilburn-Toppin F., Athanasiou A., Forrai G., Ispas M., Lesaru M., Giannotti E., Pinker-Domenig K., Van Ongeval C., Mann R.M. (2024). Misdiagnosis in breast imaging: A statement paper from European Society Breast Imaging (EUSOBI)—Part 1: The role of common errors in radiology in missed breast cancer and implications of misdiagnosis. Eur. Radiol..

[24] Thomassin-Naggara I., Athanasiou A., Kilburn-Toppin F., Forrai G., Ispas M., Lesaru M., Giannotti E., Pinker-Domenig K., Van Ongeval C., Mann R.M. (2024). Misdiagnosis in breast imaging: A statement paper from European Society Breast Imaging (EUSOBI)—Part 2: Main causes of errors in breast imaging and recommendations from European Society of Breast Imaging to limit misdiagnosis. Eur. Radiol..

[25] Geyer H.L., Viney J., Karlin N. (2010). Metastatic Carcinoid Presenting as a Breast Lesion. Curr. Oncol..

[26] Mosunjac M.B., Kochhar R., Mosunjac M.I., Lau S.K. (2004). Primary Small Bowel Carcinoid Tumor with Bilateral Breast Metastases: Report of 2 Cases with Different Clinical Presentations. Arch. Pathol. Lab. Med..

[27] Upalakalin J.N., Collins L.C., Tawa N., Parangi S. (2006). Carcinoid tumors in the breast. Am. J. Surg..

[28] Howe J.R., Cardona K., Fraker D.L., Kebebew E., Untch B.R., Wang Y.-Z., Law C.H., Liu E.H., Kim M.K., Menda Y. (2017). The Surgical Management of Small Bowel Neuroendocrine Tumors. Pancreas.

